# Evenly Distributed Microporous Structure and E7 Peptide Functionalization Synergistically Accelerate Osteogenesis and Angiogenesis in Engineered Periosteum

**DOI:** 10.1002/advs.202406084

**Published:** 2025-01-27

**Authors:** Qihong Li, Chen Li, Jun Yan, Chunli Zhang, Yu Jiang, Xiantong Hu, Liwei Han, Li Li, Peng Wang, Lingzhou Zhao, Yantao Zhao

**Affiliations:** ^1^ Department of Stomatology The Fifth Medical Center of Chinese PLA General Hospital Beijing 100071 China; ^2^ Senior Department of Orthopedics The Fourth Medical Center of Chinese PLA General Hospital Beijing 100048 China; ^3^ Xijing 986 Hospital Department The Fourth Military Medical University Xi'an 710032 China; ^4^ Beijing Engineering Research Center of Orthopedics Implants Beijing 100048 China; ^5^ Department of Neurosurgery The First Medical Center of Chinese PLA General Hospital Beijing 100853 China; ^6^ Department of Stomatology Air Force Medical Center The Fourth Military Medical University Beijing 100142 China

**Keywords:** affinitive peptide, bone regeneration, cell homing, engineered periosteum, microporous structure

## Abstract

Repairing large bone defects remains a significant clinical challenge. Stem cell is of great importance in bone regeneration, and periosteum is rich in periosteal stem cell, which has a great influence on repairing bone defects. Bioengineered periosteum with excellent biocompatibility and stem cell homing capabilities to promote bone regeneration is of great clinical significance. The E7 peptide (EPLQLKM), which exhibits a specific affinity for mesenchymal stem cells (MSCs), is beneficial for modulating cellular functions. In this study, a unique microporous structured carboxymethyl chitosan/sodium alginate membrane with a proper mass ratio is developed by the addition of Poloxam 407 （P407）, which is then functionalized with the E7 affinitive peptide. This membrane, characterized by its microporous structure and E7 peptide functionalization (CSSA/P/E), not only demonstrated favorable mechanical properties, enhanced hydrophilicity, satisfactory biodegradation profile, and excellent biocompatibility, but also synergistically enhanced MSCs recruitment. It is found to promote the proliferation, spreading, and osteogenic differentiation of MSCs in vitro and to accelerate early periosteal regeneration, bone matrix deposition, and vascularization in vivo, leading to effective regeneration of critical‐sized bone defects. Overall, this study presents a robust, cell and growth factor‐free strategy for bioengineering periosteum, offering a potential solution for the challenging large size bone defects.

## Graphical abstract

The periosteum plays a crucial role in the repair of large bone defects. The artificial periosteum is successfully engineered with a porous structure and enhanced functionality by integrating P407 (Poloxam 407) and E7 peptides. These modifications are discovered to enhance proliferation, recruitment, and osteogenic differentiation of MSCs and to promote early periosteal regeneration, bone formation, and vascularization.

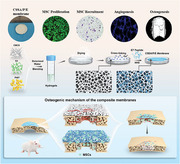

## 1 Introduction

Bone is the hard connective tissue with important function of movement supporting. Clinically, bone defect has always been a challenge for physicians and patients. The increasing incidence of bone‐related diseases with the increasing average age of the global population leads to a growing need for bone regeneration.^[^ ^1^ ^]^ Although the bone tissue possesses a high capacity for self‐regeneration, allowing most orthopedic traumas heal fully under appropriate conditions, critical‐sized bone defects caused by high‐energy trauma, disease, tumor removal, or congenital malformation cannot heal spontaneously, whose clinical interventions continue to pose big challenges.^[^ ^2^ ^]^ The bone non‐union has a prevalence ranging from 2.5 to 46%, depending on the severity of the defect and the age of the patient, and other local factors,^[^ ^3^ ^]^ which is associated with a reduced quality of life and thus surgical intervention is required to restore health and function. The conventional bone defect treatments including autologous bone graft, allogenic bone graft, and artificial bone substitute graft.^[^ ^4^ ^]^

The periosteum, a dense fibrous membrane that lines the outer surface of bones, comprises an outer fibrous layer and an inner cellular layer. It is highly vascularized and harbors an osteoprogenitor niche, serving as a reservoir for osteoblasts and stem cells, thus playing a vital role in bone development, maintenance, and remodeling.^[^ ^5^ ^]^ Research has demonstrated that autografts retaining the periosteum are superior to allografts and artificial bone grafts in repairing large segmental bone defects.^[^ ^5^, ^6^, ^7^, ^8^, ^9^, ^10^, ^11^ ^]^ Studies have also shown that removing periosteum from autogenous bone caused a 73% reduction in bone and cartilage formation and a tenfold reduction in neovascularization.^[^ ^9^ ^]^ In addition to providing blood supply and nutrition, the periosteum contributes cells, such as periosteal stem cells and osteoprogenitor cells, to participate in bone regeneration by mediating intramembranous and endochondral osteogenesis.^[^ ^5^, ^10^ ^]^ Therefore, the periosteum plays an important role in bone repair and regeneration. Designing biomaterial platforms for periosteum regeneration to induce bone therapeutics has gradually become a research hotspot. The filed focus on the construction of bioengineered periosteum by integrating bioactive molecules, growth factors, and stem cells, as well as the use of nanotechnology and 3D printing. For instance, hydroxyapatite‐nanoparticles, employed as a bioactive molecule, have been used to cross‐link alginate to fabricate a bilayered membrane that enhances osteoblast proliferation and differentiation, as well as fibroblast proliferation.^[^ ^12^ ^]^ Micro/nanofibrous bionic periosteum induced early vascularization of the fibrous membrane and bone defect area by sustained releasing of VEGF to achieve complete regeneration of periosteum and bone tissue.^[^ ^13^ ^]^ Furthermore, studies have reported that biomimetic scaffold materials assisted by external physical therapy (piezoelectric stimulation,^[^ ^14^ ^]^ near‐infrared light,^[^ ^15^ ^]^ laser irradiation^[^ ^16^ ^]^ promote immune regulation, angiogenesis, and osteogenesis (Table S1, Supporting Information).

Tissue‐engineered periosteum constructed via biomaterial sheets seeded with mesenchymal stem cells (MSCs) is also a dominant trend at present. However, this approach involves invasive donor biopsies and labor‐intensive, time‐consuming, and costly cell cultures that can also adversely affect stem cell phenotype.^[^ ^17^ ^]^ A leading strategy in tissue engineering is the design of biomimetic biomaterials that stimulate the body's repair mechanisms via homing of endogenous stem cells to the injury/defect sites, which is considered to be superior to the exogenous stem cell based approach in terms of cost‐effectiveness, minimal invasion and relative simplicity.^[^ ^18^, ^19^ ^]^ Surface modification of materials using biologically active substances should provide a promising tool to promote cell homing. Compared to functional proteins, the functional short peptides are more stable and resistant to pH and thermal changes, cost‐effective, controllable, which are characterized by reduced manufacturing cost and purification time.^[^ ^20^ ^]^ Notably, MSC affinity peptide sequences were reported to be able to specifically bind to and recruit MSCs in vitro and in vivo.^[^ ^21^ ^]^ The conjugation of the affinity peptides to biomaterials is anticipated to facilitate the recruitment and homing of MSCs from the surrounding tissue and remote site to enhance bone regeneration and integration with the host tissue. A novel E7 peptide with the amino acid sequence of EPLQLKM was demonstrated to show high specific affinity to bone marrow MSCs (BMSCs), which can recruit BMSCs and promote their adhesion.^[^ ^22^ ^]^ It can selectively capture MSCs over other cells.^[^ ^23^ ^]^ Recently, E7 has been widely used to modify biomaterials for BMSCs recruitment.^[^ ^24^ ^]^ In addition, the E7 peptide was found to promote the adhesion and proliferation of BMSCs and enhance the interaction of BMSCs with biomaterials.^[^ ^25^ ^]^ Wu et al. reported that E7 biofunctionalization activated the stromal cell‐derived factor‐1 alpha (SDF‐1α) / chemokine receptor 4 (CXCR4) axis and p38, extracellular signal‐related kinase (ERK), and Akt signal transduction pathways to regulate cell recruitment and adhesion.^[^ ^26^, ^27^ ^]^

Natural periosteum possesses a fibrous network structure and excellent affinity to cells, which are important for the growth and development of osteogenic cells.^[^ ^28^ ^]^ Hence, ideal engineered periosteum should mimic the natural extracellular matrix (ECM) structure, including fibrous/porous structure and osteogenic cell affinitive property. Microporous structures are not only essential for bone development and vascularization, but also can lower the risk of stress shielding and stiffness mismatch with natural tissue by reducing Young's modulus.^[^ ^29^ ^]^ The surface roughness generated by the microporous structures can improve cell and substrate adhesion.^[^ ^30^ ^]^ Additionally, sufficient tensile strength and mechanical stability are required to allow for surgical manipulation and to maintain the space at the bone defect site. Natural polymers and their derivatives are potential candidates for developing such bioengineered biodegradable periosteum due to their unique biocompatibility, biodegradability, and flexibility in incorporating bioactive molecules.^[^ ^31^ ^]^ As the only natural cationic polysaccharide, chitosan (CS) exhibits important biological functions such as good biodegradability, excellent biocompatibility, and antioxidant property. However, CS is only soluble in acidic solutions, presenting technical and biological challenges.^[^ ^32^ ^]^ Carboxymethyl chitosan (CMCS), as a derivative of CS, offers good water solubility in neutral solution and hence becomes an attractive alternative of CS.^[^ ^33^, ^34^ ^]^ Materials solely based on CMCS have inherent limitations, such as inadequate mechanical strength. Sodium alginate (SA) is another natural water‐soluble polysaccharide polymer derived from brown algae, which is also widely studied for bone regelation next to CS.^[^ ^35^ ^]^ Blending is a convenient and effective approach to improve the performance of polymer materials. The amino groups in CMCS show strong electrostatic interaction with SA, which improves the mechanical strength and thermal stability of CMCS‐based composites.^[^ ^36^ ^]^ CMCS/SA based composites have been used as scaffolds to promote bone regeneration.^[^ ^37^ ^]^

**Figure 1:**
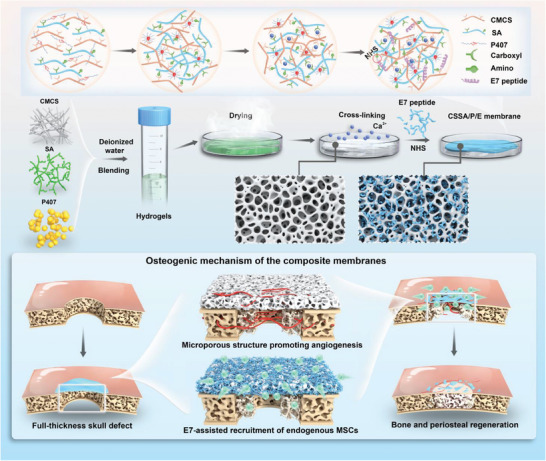
Schematic diagram of the fabrication of the artificial periosteum and its effects and mechanism of promoting bone regeneration.

In this study, a bioengineered biodegradable periosteal membrane was developed using the biodegradable polymer CMCS and SA via electrostatic interaction and cross‐linking with CaCl_2_. Poloxamers, biocompatible long‐chain polyethylene glycol‐b‐polypropylene glycol‐b‐polyethylene glycol block copolymers, are commonly used as emulsifiers, solubilizers, and wetting agents.^[^ ^38^ ^]^ Excitingly, with the addition of Poloxamer 407 (P407), which contains a hydrophilic block of poly (ethylene oxide) (PEO, 70%) and a hydrophobic block of poly (propylene oxide) (PPO, 30%) to a properly massed CMCS/SA, a specifically evenly distributed microporous structured membrane was obtained, facilitating peptide loading and MSC growth. E7 was then grafted to the membrane via NHS crosslinking, effectively promoting early recruitment of MSCs. In addition, the synergistic effect of the porous structure and E7 promoted the formation of blood vessels and periosteum in the early stage to achieve complete bone regeneration. An artificial periosteum mobilized the bone regeneration microenvironment from multiple angles to promote the formation of new bone. (**Figure** **1**).

## 2 Results and Discussion

### 2.1 Characterization of the Composite Membranes

#### 2.1.1 Surface Morphology

Both CMCS and SA have good biocompatibility, which are often used as an alternative of ECM to promote cell growth and functional differentiation in tissue regeneration. CMCS can not only promote cell adhesion and proliferation but also possess high osteoinductive capacity and accelerate calcium deposition and tissue mineralization,^[^ ^39^ ^]^ while membranes composed solely of CMCS are unsatisfactory in terms of mechanical properties. In contrast, SA displays robust mechanical strength but suffers from low protein adsorption capacity due to its strong hydrophilicity, resulting in poor cellular interaction. Consequently, in this study CMCS/SA (CSSA) composite membranes were made to achieve both satisfactory mechanical and biological behavior.

**Figure 2:**
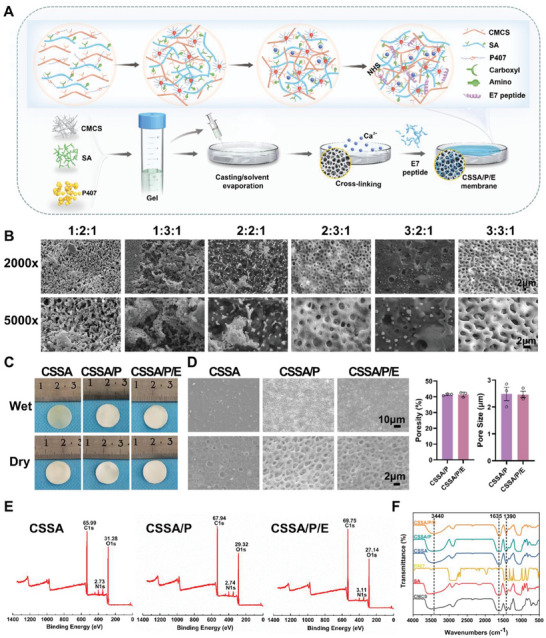
Fabrication and morphological and component analysis of different composite membranes: A) Schematic diagram for the preparation of the composite membranes; B) CSSA/P membranes formed by dissolving different mass ratios of CMCS: SA: P407 (1:2:1, 1:3:1, 2:2:1, 2:3:1, 3:2:1, and 3:3:1, where 1 portion = 100 mg) in 10 mL deionized water. The CSSA/P membrane formed with a ratio of 3:3:1 shows an evenly distributed microporous structure and clean surface; C) Macroscopic view of the composite membranes in wet and dry state; D) Representative SEM images of a relatively lower magnification (scale bars: 10 µm) and a relatively higher magnification (scale bars: 2 µm) of the composite membranes and their average pore size and porosity (n = 3); E) XPS spectra and F) FTIR spectra analysis of the composite membranes. Data are presented as mean ± standard errors of the mean (SEM). An unpaired *t*‐test was used to calculate significant significance in C.

First of all, the ideal formula of the polymer solution for the CSSA/P (CMCS/SA/P407) was screened. Membranes formed at different CMCS: SA: P407 mass ratios of 1:2:1, 1:3:1, 2:2:1, 2:3:1, 3:2:1, and 3:3:1 were examined using SEM (**Figure** **2**B). It was found that membranes at ratios of 1:2:1, 1:3:1, 2:2:1, and 3:2:1 exhibited no specific structure and contained many debris. However, membranes at the ratios of 2:3:1 and 3:3:1 demonstrated a microporous structure, with those at 3:3:1 displaying an evenly distributed microporous structure and a clean surface, which were selected for subsequent studies. P407, known for its surfactant properties, increases the solubility of CMCS and SA and can alter their shapes. The mechanism behind the formation of an even microporous structure with an appropriate mass ratio of P407 remains to be further explored. To our knowledge, this is the first report of an even microporous (CSSA/P) membrane, which may have numerous applications in the field of biomaterials.

The gross views of the composite membranes are shown in Figure 2C. In wet states, CSSA was partial translucent, while CSSA/P and CSSA/P/E (CMCS/SA/P407/E7) were white and completely opaque due to the microporous structure. But in dry states, CSSA, CSSA/P, and CSSA/P/E all appeared white and completely opaque. As shown in Figure 2D, the CSSA composite membranes, formed by the casting/solvent evaporation technique, generally exhibit a flat surface with sporadic small and shallow pores with an average diameter of < 1 µm (Figure 2D). As displayed in Figure 2B, CSSA/P shows a homogeneous microporous/fibrous structure with a pore diameter of ≈2.5 µm and the interpore struts averaging ≈ 1 µm in diameter (Figure 2D). It is evident from Figure 2D that the grafting of the E7 affinity peptide does not compromise the porous/fibrous structure of CSSA/P. The porosity of CSSA/P and CSSA/P/E is 41.40% and 41.45%, respectively (Figure 2D). A microfibrous/porous structure is reported to be beneficial for adhesion and subsequent functions of bone‐related cells.^[^ ^29^ ^]^

#### 2.1.2 FTIR and XPS Analysis

Fourier transform‐infrared spectroscopy (FTIR) and X‐ray photoelectron spectroscopy (XPS) were utilized to verify the successful grafting of the E7 affinity peptide to CSSA/P via NHS. As shown in Figure 2F, the FTIR spectra show the structural conformation of the fabricated membranes. The CMCS and SA membranes show the characteristic peaks at 3440, 1635, and 1390 cm^−1^. The absorption bands at ≈ 3440 cm^−1^ are mainly caused by the tensile vibration of O‐H and N‐H group of hydroxyl and amine groups, those at ≈ 1635 cm^−1^ are resulted from asymmetric stretching of ─COOH, and those at ≈ 1390 cm^−1^ are triggered by symmetric stretching of ‐COOH.^[^ ^4^ ^]^ Compared to the CMCS and SA membranes, the CSSA and CSSA/P membranes do not display additional FTIR peaks, but the protrusion sites of characteristic peaks shift toward right and broaden at ≈ 3440 cm^−1^. These indicate that the hydrogen bonds may play a certain role between CMCS and SA in addition to the influence of Ca^2+^. Meanwhile, compared to CSSA/P, the characteristic peaks of CSSA/P/E at 3440 and 1635 cm^−1^ become narrow, proving interactions among chemical groups. Surface functional groups of the membranes were further assessed by XPS (Figure 2E). Compared with CSSA and CSSA/P, the nitrogen peak (N1s, 399.8 eV) of CSSA/P/E increases due to the introduction of E7 peptide and NHS. In conclusion, effective chemical bonding is confirmed between the E7 peptide and NHS, and the E7 peptides are successfully grafted on the CSSA/P/E membranes through the amino groups.

#### 2.1.3 Surface Hydrophilicity

**Figure 3:**
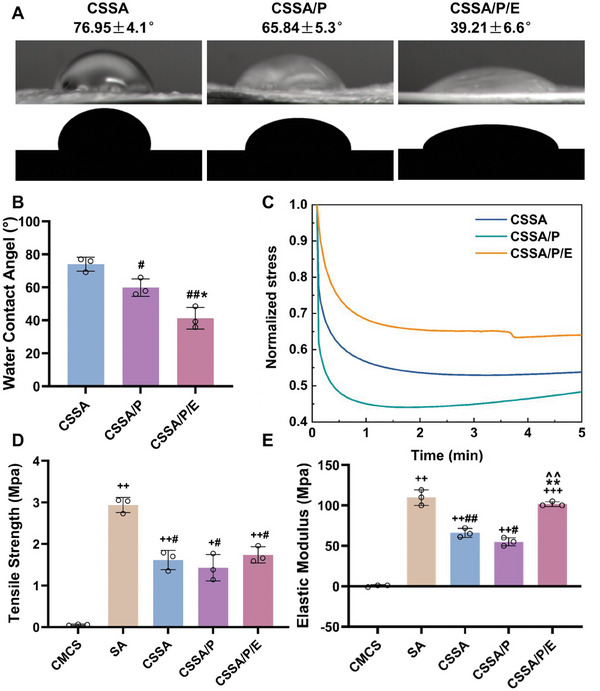
Characterization of different composite membranes: A) Contact angle assay and B) the corresponding quantitative analysis (n = 3, #*p* < 0.05 vs CSSA, ##*p* < 0.01 s CSSA, and *p < 0.05 vs CSSA/P); C) Stress relaxation of the composite membranes under wet condition; D) Tensile strength and E) elastic modulus of the composite membranes under wet condition (n = 3, +*p* < 0.05 vs CMCS, ++*p* < 0.01 vs CMCS, +++*p* < 0.001 vs CMCS, #*p* < 0.05 vs SA, ##*p* < 0.01 vs SA, and ^^*p* < 0.01 vs CSSA/P). Data are presented as mean ± SEM. Statistical significance was calculated using one‐way ANOVA in B and D‐E.

The hydrophilicity of the membranes is beneficial for cell adhesion and growth.^[^ ^40^ ^]^ Effective cell adhesion occurs on polymer surfaces with water contact angles between 40° and 70°.^[^ ^41^ ^]^ Thus, the surface hydrophilicity of the composite membranes was also assessed (**Figure** **3**A), with water contact angles recorded at 76.95 ± 4.2°, 65.84 ± 5.3°, and 54.42 ± 6.6° for CSSA, CSSA/P, and CSSA/P/E, respectively (Figure 3B). The slightly enhanced hydrophilicity of CSSA/P membranes compared to CSSA membranes may be attributed to the specific microporous structure of CSSA/P membranes. It is reported that E7 contains many hydrophilic functional groups such as amino groups, which shall be the reason for the further enhanced hydrophilicity of CSSA/P/E membranes. In line with our results, Li et al. reported enhanced hydrophilicity of biodegradable polyester after E7 peptide modification,^[^ ^25^ ^]^ and Wu et al. observed increased hydrophilicity in silk fibroin electrospun scaffolds after E7 grafting.^[^ ^42^ ^]^

#### 2.1.4 Mechanical Properties

The tensile strength and elastic modulus of the composite membranes were assessed in the wet state (Figure 3C–E). Biomimetic periosteum requires sufficient mechanical properties to withstand surgical forces and maintain space during bone defect repair/regeneration. As expected, membranes made of pure CMCS possessed extremely low tensile strength of 0.057 ± 0.015 MPa. In contrast, SA membranes displayed a significantly higher tensile strength of 2.937 ± 0.180 MPa. The tensile strength of CSSA membranes (1.613 ± 0.232 MPa) was markedly higher than that of pure CMCS membranes, proving that the addition of SA definitely improved the mechanical strength of CMCS membranes. The strong electrostatic forces and hydrogen bonds between the active amino group of CMCS and SA should lead to the close entangling of the polymer molecular chains.^[^ ^36^ ^]^ In addition, the ion exchange of Ca^2+^ with SA and the chelation of Ca^2+^ with CMCS strengthen the cross‐linked structure in the composite membranes.^[^ ^39^ ^]^ The tensile strength of CSSA/P membranes (1.430 ± 0.318 MPa) was slightly lower than that of CSSA membranes, which can be explained by the formation of the porous structure compromising mechanical strength. Interestingly, further grafting of E7 through NHS cross‐linking increased the tensile strength to 1.737 ± 0.197 MPa, surpassing that of CSSA membranes. NHS cross‐linking of the CMCS and SA can be anticipated during the E7 peptide grafting, which was reported to improve the mechanical properties, reduce the inflammatory reaction, and slow down the degradation of biomaterials.^[^ ^43^ ^]^

Elastic modulus reflects a material's ability to store energy during deformation and is a critical regulator of cell behavior, influencing how cells manipulate and interact with the biomaterial's structure. Reduced cell spreading and greater motility were observed on soft substrate compared to relatively stiffer one. In addition, the cells preferred to migrate toward stiffer regions.^[^ ^44^ ^]^ For MSCs, substrate stiffness is known to influence MSC fate, including self‐renewal and lineage differentiation.^[^ ^45^ ^]^ The general trend of elastic modulus among different membranes is similar to that of tensile strength. The elastic modulus of CMCS membranes was only 0.333 ± 1.155 MPa, potentially too soft to adequately promote MSCs spreading and subsequent osteogenic differentiation.^[^ ^46^ ^]^ On contrary, SA membranes showed much higher elastic modulus of 109.667 ± 9.504 MPa. The elastic modulus of CSSA and CSSA/P membranes was 66.000 ± 5.568 and 55.000 ± 5.000 MPa, respectively. It is noted that E7 grafting by NHS cross‐linking led to an dramatic increase in the elastic modulus to be 101.667 ± 2.887 MPa, which was similar to the value of SA. On a relatively stiffer substrate, MSCs tend to exhibit a polygonal morphology with larger spreading area, enhanced proliferation, and increased expression of osteogenic differentiation markers through a stiffness‐sensing mechanism mediated by integrin engagement and myosin‐based contraction of the cytoskeleton.^[^ ^47^ ^]^

Stress relaxation is crucial in cell‐ECM interactions. Studies have shown that the rapidly relaxing gels not only enhance the spreading and proliferation of MSCs, but also accelerate osteogenesis and strengthen the osteogenic activity of osteogenically differentiated stem cells.^[^ ^48^, ^49^ ^]^ The CSSA/P membrane showed faster and higher stress relaxation response compared to other groups, and CSSA/P/E membrane exhibited minimal stress relaxation under tensile conditions. This result may be caused by the porous structure. From this perspective, the CSSA/P membrane possessed the most substantial impact on the diffusion, proliferation and osteogenic differentiation of MSCs cells. However, the recruitment of E7 peptide in CSSA/P/E counteracts the bionic properties generated by stress relaxation.

### 2.2 In Vitro Cellular Study

#### 2.2.1 Biocompatibility

Good biocompatibility is a prerequisite for an ideal biomaterial. The CCK‐8, MTT and Live/Dead assays were conducted to evaluate the viability of MSCs on the composite membranes.

**Figure 4:**
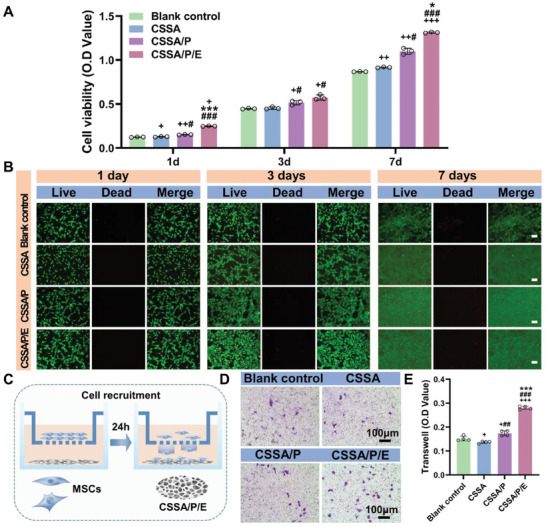
Biocompatibility and MSCs recruitment ability of the composite membranes in vitro. A) CCK‐8 assay of MSCs grown on the composite membranes and the control surface (n = 3, ++*p* < 0.01 vs control group, +++*p* < 0.001 vs control group, ##*p* < 0.01 vs CSSA, ###*p* < 0.001 vs CSSA, **p < 0.01 vs CSSA/P, and ***p < 0.001 vs CSSA/P); B) Live/dead staining of MSCs on the composite membranes and the control surface after 1, 3, and 7 days of culture (plotting scale = 100 µm); C)Schematic diagram of the composite membrane recruiting MSCs; Transwell‐migration images D) and quantitative analyses E) of MSCs recruited by the composite membranes and the control surface after 24 h of incubation. (bars = 100 µm, n = 4, +*p* < 0.05 vs control group, +++*p* < 0.001 vs control group, ##*p* < 0.01 vs CSSA, ###*p* < 0.001 vs CSSA, and ***p < 0.001 vs CSSA/P). Data are presented as mean ± SEM. Statistical significance was analyzed by one‐way ANOVA (A, E).

According to the CCK‐8 results shown in **Figure** **4**A, MSCs proliferated well on all the membranes and the control surface with time from day 1 to day 7, and the membranes induced better cell proliferation/viability compared to the cell culture plate. Given that the cell culture plate is widely recognized as biocompatible and used for in vitro studies, these results confirm the excellent biocompatibility of CSSA, CSSA/P, and CSSA/P/E membranes. At all time points, the cell viability on the composite membranes followed the trend of CSSA/P/E > CSSA/P > CSSA ≈ control, with CSSA/P/E showing the highest promotion of MSC viability and proliferation. Figure S4 (Supporting Information) showed that the MTT assay was used to evaluate the survival of MSCs on control, CSSA, CSSA/P, and CSSA/P/E membranes on the 3rd and 5th days, which was consistent with the trend of CCK‐8 results. The enhanced MSC viability/proliferation by CSSA/P compared to CSSA may be explained by the microporous structure and the higher hydrophilicity of CSSA/P. Suitable microporous structures^[^ ^50^ ^]^ and proper hydrophilicity^[^ ^51^ ^]^ are reported to promote MSC proliferation. The superior performance of CSSA/P/E in promoting MSC viability/proliferation is due to the E7 peptide, which has been shown to enhance MSC adhesion and proliferation in a dose‐dependent manner after grafting onto biomaterials.^[^ ^25^ ^]^

The Live/Dead assay results (Figure 4B) reveal that on all substrates, the vast majority of cells were alive and stained green, with only a few red‐staining dead cells. These results further illustrated the good biocompatibility of the composite membranes. Consistent with the CCK‐8 results, more cells were observed on CSSA, CSSA/P, and CSSA/P/E compared to the control, with CSSA/P/E resulting in the highest cell count. Additionally, cells exhibited larger spreading areas on CSSA, CSSA/P, and CSSA/P/E after 3 days of culture, with CSSA/P/E inducing the largest cell spreading area. Corresponding with our findings, the cell‐spreading enhancement by the E7 peptide has been previously reported.^[^ ^25^ ^]^ After 7 days of co‐culture, although more red‐staining dead cells were found compared with 1 and 3 days, possibly due to the over dense cell culture, green‐staining live cells possessed more significant increase and more dense distribution with culture time. Especially, cells in CSSA/P and CSSA/P/E groups almost cover the whole surface of the materials, further demonstrating the beneficial effects of the porous structure and E7 peptide on cell proliferation.

#### 2.2.2 Cell Recruitment

The transwell‐migration assay was conducted to investigate the migration‐inducing effects of the composite membranes on MSCs (Figure 4C). Compared to control, CSSA, and CSSA/P, CSSA/P/E recruited obviously more MSCs to the submembrane surface (Figure 4D,E), demonstrating the MSC recruiting effect of CSSA/P/E. Covalent conjugation of E7 on biomaterials has been reported to significantly enhanced the MSC recruitment in vitro^[^ ^51^ ^]^ and in vivo.^[^ ^22^ ^]^ The MSC recruiting effect of E7 may be associated with SDF‐1α/CXCR4 axis and p38, ERK, and Akt signal pathways.^[^ ^42^ ^]^

#### 2.2.3 Osteogenic Differentiation and Mineralization

**Figure 5:**
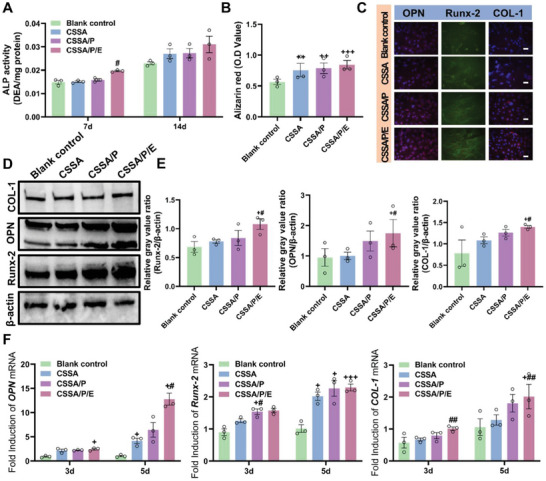
In vitro osteogenic differentiation of MSCs on the composite membranes and the control surface. A) Semiquantitative analysis of intracellular ALP activity of MSCs after 7 and 14 days of osteogenic induction (n = 3, +*p* < 0.05 vs control group); B) Quantitative analysis of calcium deposits of MSCs after 21 days of osteogenic induction (n = 3, +*p* < 0.05 vs control group); C) Immunofluorescence staining of OPN, Runx‐2, and COL‐1 in MSCs after 7 days of osteogenic induction (plotting scale = 50 µm); D,E) The western blot image and quantitative analysis of osteogenic protein expression on the composite membranes after 7 days of osteogenic induction (n = 3, +*p* < 0.05 vs control group, #*p* < 0.05 vs CSSA) F) The qRT‐PCR analysis of osteogenic gene expression on the composite membranes after 3 and 5 days of osteogenic induction (n = 3, +*p* < 0.05 vs control group, ++*p* < 0.01 vs control group, +++*p* < 0.001 vs control group, #*p* < 0.05 vs CSSA, ##*p* < 0.01 vs CSSA, and *p < 0.05 vs CSSA/P). Data are presented as mean ± SEM. Statistical significance was analyzed using one‐way ANOVA (A‐B, E‐F).

Intracellular alkaline phosphatase (ALP) activity, a marker of early osteogenic differentiation, was measured after 7 and 14 days of osteogenic induction (**Figure** **5**A). The ALP activity increased with time. On day 7, the ALP activity on CSSA/P/E was significantly higher than control. At day 14, the ALP activity showed a slimier trend to that of day 7 but without statistical difference. The narrowing difference in ALP activity between CSSA/P/E and control from day 7 to day 14 might be due to cells on CSSA/P/E advancing into the late osteoblast differentiation stage more rapidly.

Calcium nodule formation, a key indicator of late osteogenic differentiation in MSCs, was evaluated using Alizarin Red S (ARS) to assess mineral deposition after 21 days of co‐culture (Figure 5B).^[^ ^52^ ^]^ Compared to the control, CSSA, CSSA/P, and CSSA/P/E membranes produced more mineralized nodules, though no significant statistical differences in mineral deposition were observed among CSSA, CSSA/P, and CSSA/P/E, aligning with the ALP activity data.

Osteogenesis‐related gene expression was quantified using quantitative real‐time polymerase chain reaction (qRT‐PCR), with results shown in Figure 5F. The composite membranes induced higher expression levels of osteopontin (OPN), runt‐related transcription (Runx‐2), and collagen I (COL‐1) than the control. For Runx‐2, expression levels were generally similar across the composite membranes, particularly between CSSA/P and CSSA/P/E. For the expression of OPN, there was little difference among the various membranes at day 3. However, over all, the expression levels increased significantly and CSSA/P/E showed higher one than CSSA/P and CSSA at day 5. For COL‐1, on day 5 CSSA/P/E induced the highest COL‐1 expression, followed by the CSSA/P, CSSA and control group, albeit with a lesser increase. In conclusion, the expression of OPN, Runx‐2 and COL‐1 presented a trend of CSSA/P/E > CSSA/P > CSSA > control, which was consistent with the trend of western blot strips.

After 7 days of osteogenic induction, the expression of COL‐1, Runx‐2, and OPN within MSCs on the composite membranes was observed by immunofluorescence staining (Figure 5C). The composite membranes induced higher expression of OPN, Runx‐2 and COL‐1 than control. The three composite membranes induced similar expression of Runx‐2, while for OPN and COL‐1 CSSA/P/E resulted in the highest expression.

To further investigate the effect of CSSA/P/E on MSCs osteogenic differentiation, the expression levels of osteogenic proteins (Runx‐2, OPN, and COL‐1) were analyzed by Western blot after 7 days of induction, as illustrated in Figure 5D,E. Compared to control and CSSA, expression levels of Runx‐2, OPN, and COL‐1 were significantly upregulated in CSSA/P/E.

#### 2.2.4 In Vitro Angiogenesis Evaluation

**Figure 6:**
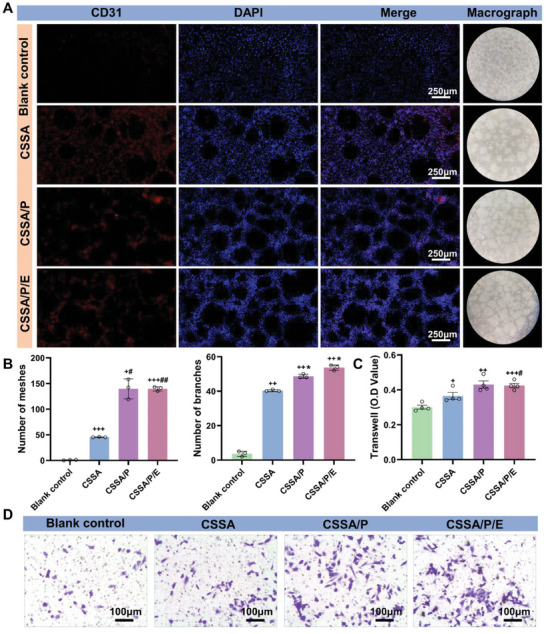
In vitro angiogenic differentiation of HUVECs induced by the samples. A) The microscopic gross and fluorescent image of tubular network formation by HUVECs in vitro (bars = 250 µm); B) Quantitative assessment of the HUVECs tube formation, including numbers of branches and meshes. (n = 3, bars = 100 µm, +*p* < 0.05 vs control group, ++*p* < 0.01 vs control group, +++*p* < 0.001 vs control group, #*p* < 0.05 vs CSSA, ##*p* < 0.01 vs CSSA, and *p < 0.05 vs CSSA/P); Transwell‐migration images D) and quantitative analyses C) of HUVECs recruited by the composite membranes and the control surface after 24 h of incubation. (n = 3, +*p* < 0.05 vs control group, ++*p* < 0.01 vs control group, +++*p* < 0.001 vs control group, ##*p* < 0.05 vs CSSA). Data are presented as mean ± SEM. Statistical significance was calculated by one‐way ANOVA in B.

Vascular network formation in the early stage of implantation is essential for bone regeneration, providing a microenvironment for cell recruitment, release of active factor, and the transport of oxygen, nutrients and metabolic waste.^[^ ^53^, ^54^ ^]^ As shown in **Figure** **6**A, the control group failed to induce tube formation after 6 h of induction, while immature tubular formation was observed in CSSA. In contrast, many mature and intact annular vessels were formed in CSSA/P and CSSA/P/E groups. Quantitative analysis of meshes and branches showed their numbers ranking as CSSA/P/E ≈ CSSA/P > CSSA > control. Additionally, subcutaneous implantation tests revealed significant early‐stage angiogenesis in CSSA/P and CSSA/P/E at 7 days post‐implantation (S 5B), further confirming the angiogenic potential of CSSA/P and CSSA/P/E. The data demonstrate the effect of the specific porous structure in inducing vascularization, and E7 peptide may have limited or no impact on early vascularization.

The transwell‐migration assay was conducted to investigate the migration‐inducing effects of the composite membranes on human umbilical vein endothelial cells (HUVECs) (Figure 6D). Compared to blank control and CSSA, CSSA/P and CSSA/P/E significantly recruited more HUVECs, which was closely related to the ordered porous structure. Moreover, no significant difference in HUVEC recruitment was observed between CSSA/P and CSSA/P/E, indicating that the E7 peptide did not significantly affect HUVEC recruitment.

### 2.3 In Vivo Osteogenesis

#### 2.3.1 Micro‐CT Evaluation

**Figure 7:**
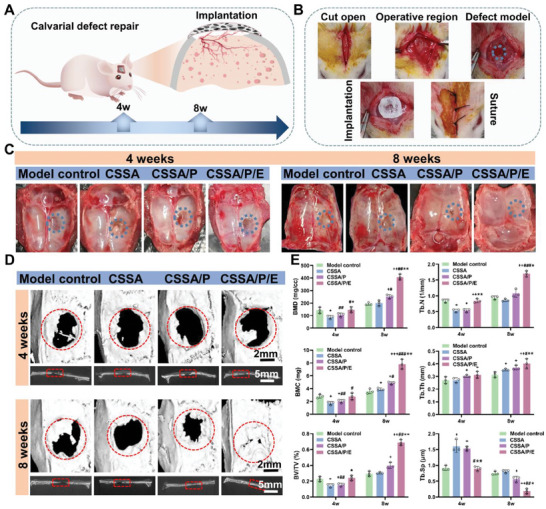
Ability of the membranes to induce in vivo new bone formation. A) Schematic diagram and B) Images for using the composite membranes to cover bone defect and the following assessments; C) Gross observation of the bone defect repair; D) Micro‐CT images of the newly formed bone at 4 and 8 weeks postsurgery (bars = 2 and 5 mm; red circles indicate the original defect area); E) The parameters analyzing the bone formation based on the micro‐CT analysis, including BMD, BMC, BV/TV, Tb.N, Tb.Sp, and Tb.Th (n = 3, +*p* < 0.05 vs control group, ++*p* < 0.01 vs control group, +++*p* < 0.001 vs control group, #*p* < 0.05 vs CSSA, ##*p* < 0.01 vs CSSA, ###*p* < 0.001 vs CSSA, *p < 0.05 vs CSSA/P, and **p < 0.01 vs CSSA/P). Data are presented as mean ± SEM. Statistical significance was calculated by one‐way ANOVA in E.

The skull samples were obtained 4 and 8 weeks after implantation of the composite membranes (**Figure** **7**B). According to gross observation, all skull specimens showed normal color without any abnormality (Figure 7C). New bone formation can be observed at the edge of the bone defect with time. Especially, after 8 weeks of healing, dramatically better healing effect could be seen in the CSSA/P/E and CSSA/P groups compared to control and CSSA. The combination of the microporous structure and E7 peptide can significantly enhance the effect of bone repair.

**Figure 8:**
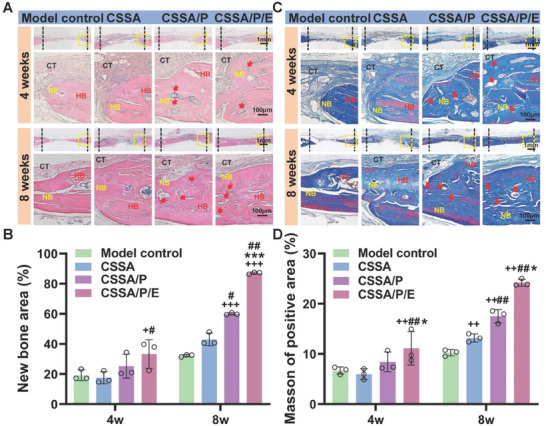
Histological analysis of new bone formed in vivo. A) H&E staining and C) Masson's Trichrome staining of new bone at 4 and 8 weeks after surgery. (bars = 1 mm and 100 µm; HB: host bone, CT: connective tissue, NB: new bone, red arrows: blood vessels, LB: lamellar bone; Black dashed line: boundary between host bone and new bone, Yellow dashed rectangle: magnified view of the defect); Quantitative analysis of the new bone area (%) based on H&E staining B) and the collagen of positive area (%) based on Masson's staining D) (n = 3). Data are presented as mean ± SEM. One‐way ANOVA was used to calculate statistical significance in B and D.

Micro‐CT images (Figure 7D) showed some newly regenerated bone at the defect edges after 4 weeks. The amount of new bone formation induced by CSSA/P/E was similar to that of the control group, while those of CSSA and CSSA/P was slightly fewer. After 8 weeks, more new bone formation was generally observed, particularly in the CSSA/P and CSSA/P/E groups. CSSA induced bone formation comparable to the control, suggesting that the simple membrane composed of CMCS and SA is ineffective to induce bone formation. CSSA/P and CSSA/P/E led to more substantial bone formation, with CSSA/P/E showing almost complete defect repair after 8 weeks, again demonstrating that the specific porous structure and the E7 peptide both have bone regeneration promoting effect. Parameters for bone formation based on Micro‐CT are detailed in **Figure** **8**E. At week 8, the parameters of bone mineral density (BMD), bone mineral content (BMC), the percentage of regenerated bone volume to total volume (BV/TV), trabecular number (Tb.N), and trabecular thickness (Tb.Th) showed the trend of CSSA/P/E > CSSA/P > CSSA > control, while the trend of trabecular separation (Tb.Sp) was opposite, revealing the higher osteogenic activity of CSSA/P/E compared to the other groups. Overall, in vivo data reveal that the membrane composed of CMCS and SA without a specific structure has no effect on bone formation, whereas that functionalized with a porous structure and E7 peptide effectively serves as an artificial periosteum to induce bone regeneration, particularly CSSA/P/E.

#### 2.3.2 Histological Evaluation

The bone regeneration was further analyzed with H&E staining. Results from H&E staining in Figure 8A showed that the bone healing progressed with the extension of healing time from 4 weeks to 8 weeks. At week 4, minimal new bone formation was observed at the edges of the bone defects across all groups. In the defect centers, there was uncalcified fibrous tissue. Compared to the control, CSSA, CSSA/P, and CSSA/P/E groups developed much thicker fibrous connective tissue. Especially, the fibrous tissue generated by CSSA/P/E was much denser and more regularly structured. After 8 weeks, a continuous layer of new bone was observed in the center of the defect, while the thickness of the new bone was different following the trend of CSSA/P/E > CSSA/P > CSSA ≈ control. Quantitative results of newly formed bone from H&E staining images were shown in Figure 8B. After 8 weeks, CSSA/P/E (86.84 ± 0.51%) had the largest area of new bone formation, with the new bone area induced by CSSA/P (60.25 ± 0.55%) exceeding that of the control (31.96 ± 0.58%) and CSSA (43.82 ± 3.11%).

Collagen deposition was assessed by Masson's trichrome staining, where newly formed bone areas were labeled in blue and host or newly matured bone appeared in red. The Masson's trichrome staining in Figure 8C showed that the amount of uncalcified fibrous tissue containing collagen showed the trend of CSSA/P/E ≈ CSSA/P > CSSA > control. Thus, the membrane coverage benefits early bone matrix deposition, with those functionalized with the microporous structure and E7 peptide performing better. From week 4 to week 8, only a slight increase in bone matrix deposition was noted for the blank control group, while significantly more bone matrix deposition was observed for the three membrane groups. At week 8, the extent of and collagen deposition and mineralization, indicated by the blue stain in Masson's trichrome staining, followed the trend of CSSA/P/E > CSSA/P > CSSA > control. CSSA/P/E led to complete repair of bone defects with highly mineralized new bone. Quantitative results of deposited collagen area based on Masson's staining were shown in Figure 8D. Eight weeks post‐implantation, the deposited collagen area of CSSA/P/E (24.21 ± 0.58%) was significantly higher than CSSA/P (17.52 ± 1.08%), CSSA (13.18 ± 0.66%), and control (10.23 ± 0.51%).

**Figure 9:**
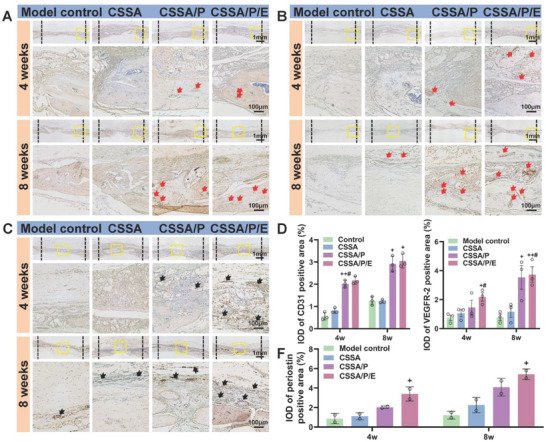
Histological analysis of new periostin and new blood vessels formed in vivo. Immunohistochemical staining of A) CD31 and B) VEGFR‐2 at 4 and 8 weeks (red arrows: blood vessel); Immunohistochemical staining of C) periostin at 4 and 8 weeks (black arrows: periosteum); D) Quantitative analysis of CD31 and VEGFR‐2 positive area; E) Quantitative analysis of periostin positive area. n = 3 samples. Bars = 1 mm and 100 µm, respectively. Data are presented as mean ± SEM. One‐way ANOVA was used to calculate statistical significance in D and F.

Early and efficient vascularization is vital for the maintenance of cell survival, active remodeling, and skeletal integrity during bone repair/regeneration. Bone engineering strategies that accelerate vascularization are promising for repairing large bone defects.^[^ ^55^ ^]^ Excitingly, H&E and Masson's staining revealed numerous blood vessels in the newly formed bone matrix in the CSSA/P and CSSA/P/E groups, while fewer were observed in the CSSA and control groups. To further verify the formation of blood vessels, immunohistochemical staining with the endothelial markers CD31 and VEGF was conducted. **Figure** **9**A,B,D shows that the presence of positive circular or elliptical rings stained with CD31 and VEGF in CSSA/P/E and CSSA/P was significantly higher than in the control and CSSA groups at weeks 4 and 8. CSSA/P/E and CSSA/P generated more new blood vessels during new bone formation, indicating that the microporous structure may play a crucial role in promoting angiogenesis.

Periostin, also known as osteoblast‐specific factor‐2 (OSF‐2), is founded in periosteum and involved in cellular function regulation,^[^ ^53^, ^56^ ^]^ and thus periostin is used as a periosteum‐specific marker. As shown in Figure 9C, CSSA/P induced more periostin expression compared to CSSA and control, and even more periostin expression was found in CSSA/P/E at both week 4 and 8. Especially, the periostin distributed evenly and continuously in the fibrous tissue over the bone defect as early as 4 weeks post‐surgery in the CSSA/P/E group, indicating its strong periosteal regeneration capability. The quantitative analysis of periostin positive area shows the trend of CSSA/P/E > CSSA/P > CSSA ≈ control (Figure 9F), suggesting that both the microporous structure and E7 peptide played crucial roles in promoting periosteal regeneration.

Collectively, the membrane functionalized with the porous structure and E7 peptide benefits early bone matrix deposition and vascularization and enhances the amount of highly mineralized new bone formation. As shown in Figure 9A,B, no cartilage matrix or fat vacuoles were observed in the histological staining, confirming that the composite membranes do not induce chondrogenesis and adipogenesis when used as engineered periosteum. Histological sections of important organs tissue including heart, liver, spleen, lung, and kidney 8 weeks after implantation showed no obvious morphological and structural changes compared to control (Figure S9A, Supporting Information), indicating that the biological safety of the composite membranes. As shown in Figure S9B,C (Supporting Information), the values of alanine aminotransferase (ALT), aspartate transaminase (AST), creatinine (Cre‐P), UREA and total bilirubin (TBIL) in each group were within a reasonable range, and the blood system was not damaged, indicating high biocompatibility of the composite membranes.

## 3 Conclusion

In the present study, the electrostatic interaction between carboxymethyl chitosan and sodium alginate, the crosslinking of Ca^2+^ and the pore‐forming effect of Poloxam 407 are utilized to prepare a composite membrane with microporous structure. Subsequently, the E7 affinity peptide was grafted onto this structure. The functionalized membrane exhibits favorable mechanical properties, enhanced hydrophilicity, satisfactory biodegradation profile, and excellent biocompatibility. Crucially, it promotes proliferation, spreading, recruitment, and osteogenic differentiation of MSCs in vitro and effectively facilitates in vivo critical‐sized bone defect regeneration through promoting early bone matrix deposition and vascularization. CSSA/P/E may constitute a robust cell and growth factor free bioengineered periosteum strategy by mobilizing the bone regeneration microenvironment from multiple angles to address clinically challenging large bone defects. Nonetheless, further studies, such as those involving large animal models and increased sample sizes, are needed to draw a conclusion on its biological effect.

## 4 Experimental Section

### Materials

CMCS (C9400) was obtained from Beijing Solarbio Science & Technology Co., Ltd. SA (LA9640) was obtained from Beijing Sunshineyeah Biotechnology Co., Ltd. P407 (P140818) was obtained from Shanghai Aladdin Biochemical Technology Co., Ltd. E7 peptide (C0724804) was obtained from Beijing Scilight Biotechnology Co., Ltd. BMSCs (mouse) were purchased from Shanghai Honsun Biological Technology Co., Ltd. DME/‐F12 (AG29749114) was purchased from Cytiva, USA. Fetal bovine serum (FBS, A4766801) was purchased from Thermo Fisher Scientific Inc., China. Penicillin‐Streptomycin‐Amphotericin B (J0408050) was purchased from M&C GENE Technology, Ltd, Beijing. Phosphate Buffered Saline (PBS, L1101300) was purchased from M&C GENE Technology, Ltd, Beijing. Cell Counting Kit‐8 (CCK‐8, SP‐BI‐C02‐2) was acquired from SUNP Biotechnology Co., Ltd, Beijing. Live & Dead Viability/Cytotoxicity Assay Kit (Calcein‐AM/PI, C2015C‐3) was acquired from Beyotime Biotechnology Co., Ltd, Shanghai. Transwell chamber (REF3422) was acquired from Costar Co., Ltd, USA. Crystal Violet Solution (0.1%, G1064) was acquired from Beijing Solarbio Science & Technology Co., Ltd. RIPA lysis buffer (P0013B) was acquired from Beyotime Biotechnology Co., Ltd, Shanghai. The p‐Nitrophenyl Phosphate (pNPP) Liquid Substrate System (C3206) was acquired from Beyotime Biotechnology Co., Ltd, Shanghai. Bicinchoninic Acid (BCA) assay (BL521A) was acquired from Beijing Labgic Technology Co., Ltd. Alizarin red S staining solution (2%, C0138) was acquired from Beyotime Biotechnology Co., Ltd, Shanghai. The rabbit monoclonal anti‐Collagen I primary antibody (COL‐1, 14695‐1‐AP) was ordered from Proteintech Group Inc., USA. And Osteopontin (OPN, RM1018) and Runt‐related transcription factor 2 (Runx‐2, D1L7F) rabbit mAb were ordered from Abcam (Shanghai) Trading Co., Ltd. Alexa Fluor 594‐conjugated secondary antibody (ab150088) was ordered from Abcam (Shanghai) Trading Co., Ltd. Trizol (REF15596018) was ordered from Life Technology Co., Ltd, USA. Glyceraldehyde phosphate dehydrogenase (GAPDH, B661304) and the primers including OPN, Runx‐2, COL‐1 were ordered from Sangon Biotech Co., Ltd, Shanghai. PrimeScript RT reagent Kit with gDNA Eraser (Perfect Real Time) (RNA‐to‐cDNA system, RR047A) was ordered from Takara Bio Inc., China. TaKaRa TB Green Premx Ex Taq II (Tli RNaseH Plus) (RR820A) was ordered from Takara Bio Inc., China. Masson trichrome staining (G1340) was obtained from Beijing Solarbio Science & Technology Co., Ltd. SABC (Rabbit IgG)‐POD Kit was obtained from Beijing Solarbio Science & Technology Co., Ltd. CD31 Polyclonal antibody (28083‐1‐AP) was purchased from Proteintech Group Inc., USA and periostin (ab315104) was purchased from Abcam (Shanghai) Trading Co., Ltd.

### Fabrication of the Composite Membranes

Composite gel membranes were prepared by the casting/solvent evaporation technique briefly described as below. The CSSA solution was prepared by dissolving 300 mg of CMCS powder and 300 mg of SA powder in 10 mL deionized water with stirring and ultrasonic vibration (KQ‐100DE, Kun Shan Shu Mei, China) for 3 h. Similarly, the CSSA/P solution was formed by dissolving different mass ratios of CMCS powder: SA powder:P407 (1:2:1, 1:3:1, 2:2:1, 2:3:1, 3:2:1, 3:3:1, where 1 portion = 100 mg) in 10 mL deionized water. After centrifuging at 5000 rpm for 5 min to remove bubbles, the solutions of 6 mL were cast into 60 mm polyethylene petri dishes, dried for 2 h at 37 °C, cross‐linked with 0.5 m CaCl_2_ solution for 30 min, and finally washed with deionized water to remove unbound Ca^2+^ to generate the CSSA and CSSA/P composite gel membranes. Afterward, the CSSA and CSSA/P membranes were prefrozen at −80 °C for 30 min and then freeze‐dried for 24 h. It was found that the CSSA/P membranes formed with 300 mg CMCS powder, 300 mg SA powder, and 100 mg P407 showed ideal microporous structure (Figure 2B), which was chosen for the following study. The E7‐covalently bound CSSA/P (CSSA/P/E) was obtained by immersion CSSA/P in 1% NHS for 12 h and then in 0.2 mg mL^−1^ E7 solution for 12 h. Afterwards, the unbound E7 peptides were removed by immersion in fresh deionized water for 6 h four times. Afterwards, CSSA, CSSA/P, and CSSA/P/E were prefrozen at −80 °C for 30 min and then freeze‐dried (FD8‐3a, SIM International Group, USA) for 24 h. The composite membranes were sterilized with Co‐60 irradiation at 25 kGy.

### Characterization of the Composite Membranes

For the structure and compositions of membrane, surface morphology and pore size of the membranes were observed under a scanning electron microscopy (SEM, SUPRA55, ZEISS, Germany). Briefly, the membranes were fixed on the sample stub of the SEM with conductive tapes, coated with gold using a gold sputtering device, and then examined. Meanwhile, FTIR, TENSOR 27, BRUKER, Germany was utilized to analyze the functional groups in the membranes. X‐ray photoelectron spectroscopy (XPS, K‐Alpha, Thermo Fisher Scientific, China) was carried out to analyze the chemical elements. Electromechanical universal testing machine (Z50, Zwick‐Roell, Germany) was used to examine the tensile strength of the membranes. Experiments were conducted with n = 3 samples. Maximum load and deformation were recorded, and tensile strength and elastic modulus were calculated. Dynamic mechanical analysis (DMA, TA Q800, America) evaluated the stress relaxation properties of rectangular‐shaped tensile‐molded samples measuring 20 mm × 5 mm × 2 mm. The hydrogel samples were performed with a strain of 3%, a temperature of 37 °C, and an amplitude of 30 µm, and the relaxation modulus was recorded with time up to 5 min. For the measurements of surface hydrohilicity, the water contact angle (JC2000DM, ZYKX, Beijing) was used to assess the surface hydrophilicity of the membranes. Membrane samples measuring 1 cm × 1 cm were placed on the stage of the contact angle meter. A charge‐coupled device camera was used to capture images of the droplets, and the device's built‐in software was applied to analyze the results. The value was the average of three experiments.

### In Vitro Cytocompatibility

CCK‐8 assay, MTT assay and live/dead cell staining assay were used to evaluate the cytocompatibility of the composite membranes. In the CCK‐8 assay, sterilized membranes were cut into disks of 6 mm in diameter and put in the 96‐well plates to rehydrate in 100 µL serum‐free DME/F12 for 30 min. After removing the serum‐free medium, 100 µL MSCs suspension of 5×10^4^ cells mL^−1^ was seeded on each composite membrane and cultured at 37 °C in an incubator with 5% CO_2_ for 1, 3, and 5 days. The tissue culture plate was applied as control. At predetermined time points, the culture media was drained, and the samples were washed with PBS. Then 100 µL 10% CCK‐8 solution was added to each well to incubate for 1 h at 37 °C. A microplate reader (357‐808025, Thermo Fisher Scientific, China) was used to determine the optical densities (ODs) at 450 nm. In the live/dead cell staining assay, sterilized membranes were cut into disks of 14 mm in diameter and put in the 24‐well plates. After 30 min of rehydration in DME/F12, 1 mL MSCs suspension of 2×10^4^ cells mL^−1^ was seeded on each composite membrane and cultured at 37 °C in an incubator with 5% CO_2_ for 1, 3, and 7 days. After removing the media and washing with PBS, 250 µL of mixed staining solution of calcein‐acetoxymethyl (AM) and propidium iodide (PI) was introduced to each well and incubated in the dark for 30 min at 37 °C. The cytoplasm of viable cells was stained into green with calcein‐AM, while the nuclei of non‐viable cells was stained in red by PI. Laser scanning confocal microscopy (IX‐2‐UCB‐2, OLYMPUS CORPORATION, Tokyo) was used to observe the fluorescence staining.

### In Vitro Cell Recruitment

The recruitment of MSCs and HUVECs by the composite membranes was measured by a transwell‐migration assay. Sterilized membranes (disks of 14 mm in diameter) were put in the lower chamber. After 30 min of rehydration, 200 µL MSCs or HUVECs suspension of 2 × 10^5^ cells mL^−1^ was seeded in the transwell upper chamber. After 24 h of culture, the transwell chamber was fixed in 4% paraformaldehyde for 20 min and stained with 0.1% crystal violet for 20 min in the dark. MSCs or HUVECs that did not penetrate the filter were wiped off with cotton swabs. Migrated cells to the lower surface of the filters were observed under a microscope (Axiolab 5, Precise Instrument, Beijing) and semi‐quantified by detecting the ODs at 450 nm after destaining in 33% acetic acid.

### In Vitro Osteogenic Activity

The membranes (disks of 14 mm in diameter) were put in the 24‐well plates to first rehydrate for 30 min. Then 1 mL MSCs suspension with 2 × 10^4^ cells was seeded on each composite membrane and kept at 37 °C in an incubator with 5% CO_2_. The culture medium was replaced every 2 days. After 7 and 14 days, the samples were washed in PBS, treated with lysis buffer, and the supernatants were collected. The ALP activity was measured with the pNPP liquid substrate system. Meanwhile, 10 µL of lysis solution was used to measure the protein concentration by a BCA protein assay. Finally, the relative ALP activity was calculated based on the results of both.

After 21 days of culture, Alizarin red S staining (ARS) was applied to analyze the formation of calcium nodules. MSCs that co‐cultured with the membranes were washed twice with PBS for 5 min each, fixed with 4% paraformaldehyde solution for 15 min, and washed again with PBS. Then 2% Alizarin red S staining was used to stain the membranes 30 min at room temperature. After washing in PBS, 10% cetylpyridinium chloride was added to dissolve the dye and to measure the absorbance at 570 nm using the microplate reader for quantitative analysis.

After 7 days of culture, MSCs on the membranes were fixed in 4% paraformaldehyde solution for 5 min and then washed in PBS. Immunofluorescent staining for bone‐specific proteins OPN, Runx‐2, and COL‐1 was performed using rabbit monoclonal anti‐OPN, anti‐RUNX2, and anti‐COL‐1 primary antibodies and Alexa Fluor 594‐conjugated secondary antibodies. The fluorescence intensity was observed under a fluorescence microscope to evaluate the osteogenic ability.

After 3 and 5 days of culture, the gene expression was analyzed by quantitative real‐time polymerase chain reaction (qRT‐PCR, REF‐A28134, Life Technologies, Singapore). Briefly, total RNA was extracted from MSCs on the membranes using Trizol, and 2 mg of RNA from each sample was reverse transcribed into complementary DNA (cDNA) using an RNA‐to‐cDNA master mix kit. Expression levels of genes including OPN, Runx‐2, and COL‐1 were quantified using TaKaRa TB Green Premx Ex Taq II. Gene expression data were analyzed by the 2^−ΔΔCT^ method, normalized to the GAPDH expression data and expressed as fold ratio of the blank control group. The primers used were listed in **Table** **1**.

**Table 1:** Primers used for qRT‐PCR assay.

Gene	Sequence (5′‐3′)
GAPD‐hF	TGGTGAAGCAGGCATCTGAG
GAPD‐hR	TGAAGTCGCAGGAGACAACC
COL‐1‐hF	TTCGTGACCGTGACCTTGAG
COL‐1‐hR	TCTCCGCTCTTCCAGTCAGA
Runx‐2‐hF	CCTCAGTGATTTAGGGCGCA
Runx‐2‐hR	ACTTGGTGCAGAGTTCAGGG
OPN‐hF	CTTTACAGCCTGCACCCAGA
OPN‐hR	TTCTGTGGCGCAAGGAGATT

After 7 days of culture, the osteogenesis‐related protein expression was evaluated by Western blot. Total protein of the MSCs grown on the membranes was extracted by RIPA buffer with 1% protease inhibitor. The protein concentration was quantified using a BCA protein kit. After being denatured at 95 °C for 5 min, protein samples were separated by SDS‐PAGE (Electrophoresis System, PowerPac HC, American) on 4–20% Precast PAGE gel and transferred onto polyvinylidene fluoride (PVDF) membranes by a wet transfer method. Then, the PVDF membrane was blocked with 5% non‐fat milk for 1 h at room temperature, washed three times with PBS‐TWEEN‐20, and incubated overnight at 4 °C with primary antibodies including including anti‐β‐actin (1:2000), anti‐OPN (1:2000), anti‐RUNX2 (1:1000) and anti‐COL‐1 (1:2000). After washing, the membrane was incubated with goat anti‐rabbit secondary antibodies for 1 h at room temperature, developed using enhanced chemiluminescence (ECL), and imaged with a chemiluminescence imager (Tanon‐5200Multi, China). Subsequently, the protein bands of Western blot images were semi‐quantitatively measured by Image J software and the relative gray values were analyzed by Graphpad software.

### In Vitro Angiogenesis Ability

Tube formation ability of the engineered periosteum on human umbilical vein endothelial cells (HUVECs) was assessed using Matrigel basement membrane matrix as a substrate material in vitro. Pre‐thawed Matrigel was added to precooled 24‐well plate at 150 µL/well and incubated at 37 °C for 30 min to polymerize. The well‐grown HUVECs were digested with 0.25% pancreatin and resuspended in serum‐free medium. The cells were seeded onto the Matrigel at a concentration of 3 × 10^5^ cells/well and cultured with the extract solution of control, CSSA, CSSA/P and CSSA/P/E composite membranes. After incubation for 6 h, a light microscope was used to observe the tubular formation. Subsequently, the cells were fixed using 4% paraformaldehyde solution for 5 min and stained by immunofluorescence with CD31 antibody and DAPI. Images were captured with a Panoramic digital scanner (NanoZoomer S60, Japan), and the number of meshes and branches formed was analyzed by Image J software.

### Evaluation of In Vivo Performance

Totally 36 healthy male SD rats weighing 200 ± 20 g were included in this study. All animals operations were approved by the Institutional Animal Care and Use Committee of the SPF (Beijing) Biotechnology Co., Ltd (Approval No. AWE2022030401). These rats were randomly divided into four groups to receive CSSA, CSSA/P, or CSSA/P/E or as blank control (three rats per group at random). All experimental animals were kept in the animal experiment center with free access to standard laboratory diet and water. A calvarial defect model in rats was employed in this study. The rats were anesthetized by intraperitoneal injection of 0.7 mL 3% pentobarbital solution. After fixing on the operating table and removing the hair at the surgical area with electric razor, the surgical area was wiped with iodophor for disinfection. A midline incision was made from the nasal bone to the posterior nuchal line. A partial thickness flap was done and the periosteum overlying and around the defect was removed with a blade. After the cranial bone being completely exposed, a 5 mm diameter round full‐thickness defect was made at the center of left cranial bone utilizing a trephine bur and cooled continuously by 0.9% saline solution irrigation. CSSA, CSSA/P, and CSSA/P/E circular membranes, each 10 mm in diameter, were placed to completely cover the defect. Rats in the blank control group underwent the same operation but without membrane implantation. The wound was closed using 4‐0 resorbable sutures.

The rats were euthanized with CO_2_ to harvest the skulls with soft tissue 4 and 8 weeks after surgery for Micro‐CT and histological analysis. Micro‐CT scanner (GE e Xplore Locus, USA) was applied to scan the skulls with the parameters of a scanning resolution of 48 µm, a rotation angle of 360°, and a voltage of 80 kV. The obtained CT images were reconstructed to 3D images by the built‐in software. Cylindrical regions measuring 0.8 mm in height and 5 mm in diameter at the defect areas were designated as the areas of interest. Within these regions, the bone mineral density (BMD), bone mineral content (BMC), the percentage of regenerated bone volume (BV) to total volume (TV), trabecular number (Tb.N), trabecular separation (Tb.Sp), and trabecular thickness (Tb.Th) were quantitatively analyzed. For histological analysis, the harvested samples were fixed with 4% polyformaldehyde overnight and decalcified at 37 °C in a shaker (ZWY‐200B, Zhongyi Guoke, Beijing) in 10% EDTA, which was replaced once every week. After 5 weeks, the samples were embedded in paraffin and sectioned into 6‐µm thick slices through the center of circular defects. Three conseccutive sections were produced and fixed on slides. After spreading, baking (Water Bath‐Slide Drier, PHY‐III, ZONWAY, Changshan), and dewaxing, the H&E staining and Masson trichrome staining were performed. Moreover, to evaluate the expression of CD31, VEGFR‐2 and periostin, SABC (Rabbit IgG)‐POD Kit was applied for immunohistochemical staining. Histological analysis was conducted to observe the situation of bone repair and angiogenesis under a light microscope.

### Statistical Analysis

Data were expressed as mean ± SEM from at least three independent experiments. Images were randomly obtained from the samples. Statistical analyses were performed using the GraphPad Prism 8.0 software package and Origin 2018 software package. The experimental data were evaluated using one‐way ANOVA for multiple group comparisons and an unpaired Student's t‐test for two‐group comparisons. A *p*‐value of less than 0.05 was considered statistically significant.

## Conflict of Interest

The authors declare no conflict of interest.

## Supporting information



Supporting Information

## Data Availability

The data that support the findings of this study are available from the corresponding author upon reasonable request.
