# Supplementary material for: Evenly Distributed Microporous Structure and E7 Peptide Functionalization Synergistically Accelerate Osteogenesis and Angiogenesis in Engineered Periosteum
Source: Adv Sci (Weinh). 2025 Jan 27;12(11):2406084. doi: 10.1002/advs.202406084 (PMC11923966; doi:10.1002/advs.202406084)
Supplement: Supplementary file 1 — Supporting Information [file ADVS-12-2406084-s001.docx]

**Evenly Distributed Microporous Structure and E7 Peptide Functionalization Synergistically Accelerate Osteogenesis and Angiogenesis in Engineered Periosteum**

Qihong Li^1#^, Chen Li^1,2#^, Jun Yan^4#^, Chunli Zhang^2,3^, Yu Jiang^2,3^, Xiantong Hu^2,3^, Liwei Han^2,3^, Li Li^2,3*^, Peng Wang^5*^, Lingzhou Zhao^6*^, Yantao Zhao^2,3*^

1. Department of Stomatology, The Fifth Medical Center of Chinese PLA General Hospital, Beijing 100071, China.

2. Senior Department of Orthopedics, The Fourth Medical Center of Chinese PLA General Hospital, Beijing 100048, China.

3. Beijing Engineering Research Center of Orthopedics Implants, Beijing 100048, China.

4. Xijing 986 Hospital Department, The Fourth Military Medical University, Xi'an 710032, China.

5. Department of Neurosurgery, The First Medical Center of Chinese PLA General Hospital, Beijing, 100853, China.

6. Department of Stomatology, Air Force Medical Center, The Fourth Military Medical University, Beijing 100142, China.

*Correspondence: Yantao Zhao (biodoctor1981@163.com); Lingzhou Zhao (zhaolingzhou1983@hotmail.com); Peng Wang (wangpeng301@foxmail.com); Li Li (lili304@126.com).

**1.Table S1. The research on artificial periosteum.**

| Year | Team | Materials and technology | Types | Advantage | Disadvantage |
| --- | --- | --- | --- | --- | --- |
| 2020 | Noelia L. D’Elía et al | Nanohydroxyapatite (HAn), alginate | bioactive molecules; nanotechnology | Bilayered membrane can guide bone regeneration on the side and promote wound healing on the other side | It simply regulates osteoblast proliferation to promote bone.  In vivo experiment was not performed |
| 2019 | Liang Wu et al | Self-assembly of collagen together with electrospun fibers | growth factors; nanotechnology | Hierarchical micronanostructures simulate early vascularization by the sustained releasing of VEGF to achieve complete regeneration of periosteum and bone tissue. | It simply regulate vascularization to promote bone |
| 2023 | Huifan Liu et al | Piezoelectric;  PHBV, PHA (antioxidized polydopamine-modified hydroxyapatite), PBT | bioactive molecules; | Bioinspired piezoelectric periosteum  induced M2 macrophage polarization and promote the adhesion, proliferation, spreading and osteogenesis of MSCs. | Additional adjuvant therapy in vitro is required |
| 2024 | Minhao Wu et al | Near-infrared (NIR) photothermal;  DFO-loaded BPPD | bioactive molecules; | Promote bone formation and angiogenesis;  Eliminate excessive reactive oxygen species;  Induce macrophage polarization to M2 phenotype;  Possess excellent NIR/pH dual-responsive properties. |
| 2019 | Jiannan Wu et al | Electrospun fibers;  SF PDA | bioactive molecules; stem cells | E7 highly recruits BMSC to induce bone regeneration | The effect of the material on blood vessels has not been further studied. |

**2. Grafting rate and release rate assay**

Fluorescent E7 peptite (Scilight Biotechnology, China) was used to detect the grafting rate of E7 on CSSA/P membranes. The membranes were first immersed in a 1% NHS solution for 12 h, then placed in a 0.2 mg/mL fluorescent E7 solution and incubated at 4°C. The fluorescent E7 solution was taken at 2, 4, 6, 8, 10 and 12 h, respectively, and the fluorescence intensity was detected by multifunctional enzyme-labeled instrument. A percentage curve was prepared to assess the grafting rate of fluorescent E7 peptide onto the CSSA/P membranes. After immersion in the fluorescent E7 solution, the CSSA/P/E membranes were transferred into PBS and placed in a horizontal shaker at room temperature. The absorbance of the fluorescent E7 solution was measured at 1, 3, 5, 7, 9, 11, 13, and 15 days. At the same predesignated time, fluorescence electron microscopy was used to capture the fluorescence signal on the CSSA/P/E membranes. The release kinetics of E7 peptide was assessed by a percentage curve and fluorescence image.

**Results analysis**

Grafting curves were plotted to verify the grafting rate of E7. As shown in Figure S1A, the grafting rate of E7 increased with immersion time and plateaued at 8 hours, reaching 21.43±3.92%. The amount of E7 grafted per membrane was calculated to be 0.04 mg. This is consistent with or even superior to previous studies. Wentao Zhang developed a hydrogel membrane for stem cell recruitment that incorporated E7 peptide, with a grafting amount of 0.006 mg [1]. Qian Li prepared biodegradable polyester modified with E7 peptide, achieving grafting rates of 5.8%, 14.2%, and 23.8% [2].

The release kinetics of the E7 peptide were designed to determine its release rate. As shown in Figure S1B, 19.18±0.99% of the E7 peptide was released on the first day, with complete release occurring around the seventh day. Moreover, as shown in Figure S1C, the fluorescence intensity on the material gradually weakened over time and stabilized after 7 days, which further supported the quantitative analysis. Despite the early release of E7, the recruitment of MSCs was unaffected, as a large number of MSCs have been recruited in vivo at 7 days to participate in subsequent osteogenesis (Figure S8).


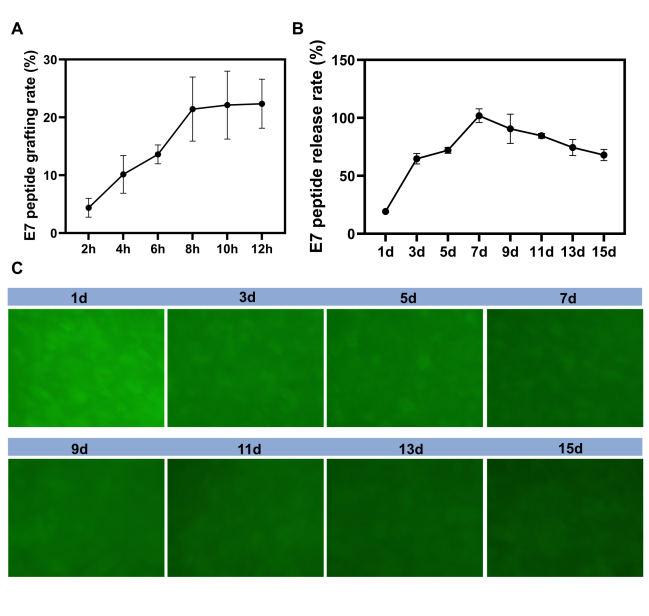


**Figure S1**. E7 peptide grafting rate (A) and release rate (B) of composite membranes; (C) Images of the artificial periosteum after releasing fluorescent E7 peptide within 15 days.

**3. Morphological change of the composite membranes after stretching**


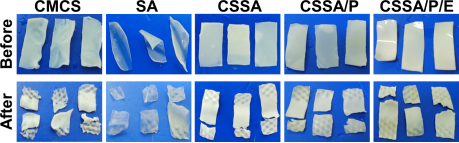


**Figure S2**. Morphological change of the composite membranes after stretching.

**4. MSCs identification**

MSCs were identified by flow cytometry and three-line differentiation. When the third generation of MSCs grew to 80%-100%, they were detached with 0.25% Trypsin-EDTA solution and resuspended in PBS at a concentration of 1 × 10^6^ cells/tube. Positive antibodies (CD45, CD34) and negative antibodies (CD29, CD90) labeled with PE or Cy7 were added to the cells suspension, respectively, incubated at room temperature for 30 min in the dark. Following incubation, samples were centrifuged and washed three times with PBS to remove the residual antibodies, and cells were identified by flow cytometry. Concurrently, MSCs in 6-well plates were induced with osteogenic (10 nM dexamethasone, 0.05mM ascorbic acid, 10mM β-glycerophosphate), lipogenic (10 nM dexamethasone, 0.5 mM IBMX, 50 mM indomethacin, 0.01 mg/mL insulin, 50 ug/mL ascorbic acid) and chondrogenic (OriCell, Rat bone marrow mesenchymal stem cell chondrogenic differentiation kit) media. After approximately 20 days, alizarin red, oil red O, and Alcian blue staining were used to evaluate osteogenic, chondrogenic and andipogenic differentiation, respectively.

**Results analysis**

In order to verify the multi-direction differentiation capability, well-grown cells were induced toward lipogenesis, osteogenesis, and chondrogenesis. As shown in Figure S3A, red lipid droplets were observed in MSCs stained with oil red O, red nodules of calcium deposition were observed in MSCs stained with alizarin red, and the cartilage was stained with wathet blue. In addition, flow cytometry (Figure S3B) showed that the cells were positive for CD90 and CD29 while negative for CD45 and CD34. The data verified the MSC nature of the cells used in the present study.


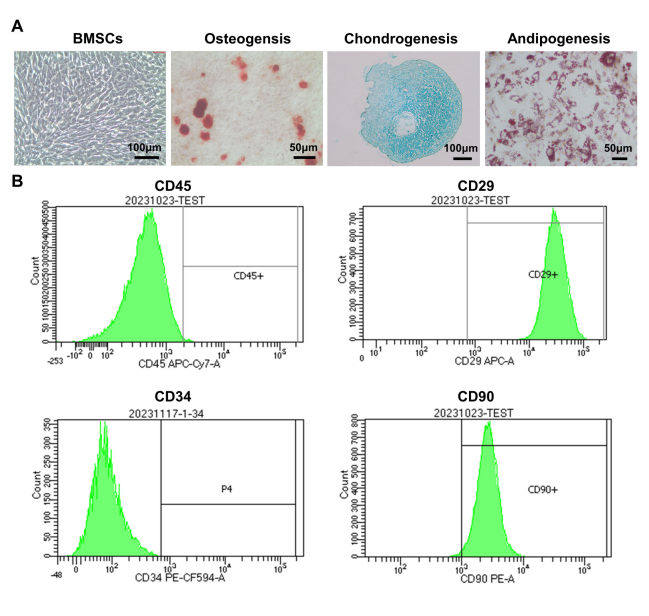


**Figure S3**. Identification of MSCs: (A) The general observation of MSCs cultured to the third generation and representative image Alizarin red staining, Oil red staining, and Alcian blue staining (bars = 100 μm or 50 μm); (B) Surface antigens (CD45, CD34, CD29, and CD90) detected by flow cytometry.

**5. *In vitro c*ell toxicity**

In the MTT assay, following a 3-day and 5-day co-culturing period, the medium was removed and cleaned with PBS. Then each well was added with 200 μL the DMEM/F12 medium containing 10% MTT (5 mg/mL) reagent and incubated at 37℃ for 4 h. At the end of incubation, the resulting solution was substituted with 150 μL of DMSO and incubated for 10 min in shock to solubilize formaldehyde crystals and develop color. Subsequently, the absorbance values of each well was measured using a microplate reader at a wavelength of 490 nm.

**Results analysis**

To further verify biocompatibility of artificial periosteum, MTT was used to evaluate the viability of MSCs on the composite membranes. Figure S4 showed that the MTT assay was used to evalute the survival of MSCs on control, CSSA, CSSA/P, and CSSA/P/E composite membranes on the 3rd and 5th days, which was consistent with the trend observed in CCK-8 results.


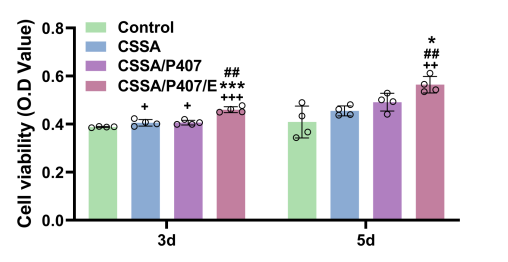


**Figure S4**. MTT assay of MSCs grown on the composite membranes and the control surface (n = 4, +*p* < 0.05 vs control group, +++*p* < 0.001 vs control group, ##*p* < 0.01 vs CSSA, ###*p* < 0.001 vs CSSA, **p* < 0.05vs CSSA/P, and ****p* < 0.001 vs CSSA/P). Data are presented as mean ± SEM. One-way ANOVA was used to calculate statistical signiﬁcance.

**6. *In vitro and vivo* degradation analysis**

The biodegradation properties of the membranes were investigated by measuring the remaining weight after immsersion in simulated body fluid (SBF), whose initial value of pH was adjusted to 7.4 by HCl and NaOH solution. Circular membranes (d =1.5 cm) were placed in tubes with 17.5 mL of SBF and maintained in a water bath at 37 ^o^C. The SBF solution was refreshed every two days to prevent saturation. The pH of the degradation liquid was monitored using a pH meter (FiveEasy Plus™, METTLER TOLEDO, China). The remaining membranes were taken out from the tubes after 7, 14, 21, 28, and 35 days, which were dried at 37℃ for 2 h to a constant weight (W_1_). Experiments were conducted with n = 3 samples. The weight remaining percentage (WR%) was calculated via Equation (1), where W_0_ is the original mass of the membranes.

WR% = W_1_/W_0_ × 100% (1)

The animal experiments were approved by the Institutional Animal Care and Use Committee of the SPF (Beijing) Biotechnology Co., Ltd (Approval No. AWE2022030401). All experimental animals were kept in the animal experiment center with free access to standard laboratory diet and water.

Subcutaneous implantation in rats was performed using eighteen healthy male SD rats (weight 200±20g), divided randomly into three groups to receive CSSA, CSSA/P, or CSSA/P/E (six rats per group). The rats were anesthetized by intraperitoneal injection of 0.7 mL 3% pentobarbital solution. After being secured to the operating table, the hair on the dorsal sides was trimmed with an electric razor, and the surgical area was wiped with iodophor for disinfection. Incisions approximately 1.5 cm long were made on both sides of the spine, and a subcutaneous pocket about 1.5 cm in diameter was created using hemostatic forceps. Membranes (14 mm in diameter) were gently inserted into the subcutaneous space with forceps, and the incisions was sutured with 4-0 absorbable sutures. The rats were sacrificed 7 and 14 days post-surgery for histological analysis.

The specimens were fixed with 4% paraformaldehyde for 24 h, dehydrated by dehydrator (ASP300S, Leica, Shanghai), embedded in paraffin (Paraffin Embedding Station, EG1150H, Leica, Switzerland), and sectioned into 6 μm thick slices (Microtome, RM2165, Leica, Switzerland). Then hematoxylin and eosin (H&E) staining was used to observe the residues of membranes and the growth of surrounding tissues.

**Results analysis**

The degradation rate and the influence on the *in vivo* microenvironment are important factors to consider for biomaterials. The biodegradation of the composite membranes was tested by WR%. Besides, the pH change of microenvironment was monitored. Over time, the remaining mass of the composite membranes decreased, and the degradation rate increased rapidly from day 21 to day 28. It is noted that CSSA, CSSA/P, and CSSA/P/E showed great stability during the first 21 days and degraded very little (Figure S5A-B). The quantitative results were consistent with the gross observation. At day 21, the WR% values of CSSA, CSSA/P, CSSA/P/E were 93.68%, 97.29%, and 91.76%, respectively. After 21 days, all the membranes showed relatively faster degradation profiles. By day 35, the WR% values of CSSA, CSSA/P, and CSSA/P/E were 48.78%, 57.69%, and 65.00%, respectively. The higher WR% of CSSA/P compared to CSSA may be explained by the porous structure of CSSA/P that allows more extensive cross-linking by Ca^2+^. The further higher WR% of CSSA/P/E compared to CSSA/P can be attributed to the NHS cross-linking during E7 peptide grafting. The relatively slower biodegradation profile of CSSA/P/E is anticipated to better meet clinical needs for artificial periosteum. In the initial weeks post-surgery, good mechanical property and integrity for the artificial periosteum are required to maintain the underlying bone defect space, prevent colonization by rapidly proliferating epithelium and soft connective tissue, allow growth of slower-growing bone-related cells, and support new bone connective tissue development. Eventually, the artificial periosteum shall degrade gradually to be replaced by the newly formed tissues, including the periosteum.

The acid-base balance within the bone microenvironment is important for the remodeling process and the interaction between osteoblast and osteoclast. It has been postulated that a relatively high local pH is necessary during the bone formation process, since the ALP activity increases at pH 8.5 in comparison with the nominal “physiological” value of 7.4 [3]. Disappointedly, all composite membranes caused a slight decrease in pH to approximately 6.4 after 7 days, which remained relatively stable until day 21. Subsequently, the pH slightly increased to about 6.8 from day 21 to day 28 and then stabilized at this level (Figure S5F). The cause of the pH decrease is unclear and requires further investigation. Fortunately, subsequent studies demonstrated that the pH decrease during degradation have no influence on the *in vitro* biocompatibility and *in vivo* bone formation. The pH change during the membrane degradation is within the acceptable range, as Hazehara-Kunitom found that acid preconditioning (pH 6.8) not only enhanced the expression of stem cell markers on MSCs, but also improved cell viability and proliferation [4]. A material’s microenvironment pH *in vivo* may be affected by a cascade of tissue responses on the biomaterial surface (e.g. plasma protein binding, coagulation, and immune response) and can be neutralized by blood and tissue fluid perfusion [3]. The body maintains pH stability by regulating the balance of carbon dioxide, bicarbonate and H^+^ through respiration, renal excretion, and bone buffering [5]. In addition, pH-stabilizing elements may be incorporated into the membranes to counter the release of acidic oligomers/monomers during degradation [6].

The ideal degradation rate of biodegradable materials should be consistent with the speed of bone healing. Moreover, both the material and its degradation products must exhibit excellent biocompatibility. The *in vivo* biodegradation of the membranes was inspected through the subcutaneous implantation experiment. From the gross picture (Figure S5C), with the elongation of implantation time from 7 to 14 days, the areas containing the membrane implants become smaller, indicating the gradual degradation of the membranes *in vivo*. No obvious sign of inflammation was observed, indicating the favorable biocompatibility of the membranes during degradation. Excitingly, significant vascularization was induced by CSSA/P and CSSA/P/E, whereas CSSA alone did not induce such changes. Moreover, CSSA/P/E led to enhanced vascularization compared to CSSA/P. These results indicate that the membranes’ specific porous structure and the E7 peptide may both have the effect to induce vascularization. The ability of CSSA/P/E to promote vascularization shall benefit its bone repairing effect, as early and effective vascularization is crucial for bone healing.

In the H&E stained images (Figure S5E), the membranes became incomplete at day 7, and by day 14, only fragments remained, proving the gradual degradation of the membranes over time. Reactive capsules can be found around the membranes. The implantation of a foreign materials will trigger an inflammatory response called foreign body reaction (FBR) [1], with most biomaterials initiating varying degrees of FBR [2]. Generally, biodegradable biomaterials may induce a higher degree of FBR due to the degradation products. The typical stages of the FBR include rapid initial protein adsorption, the recruitment and induction of various cells such as monocytes/macrophages, foreign body giant cells, inflammatory cells, wound healing cells, and extracellular matrix formation, which leads to fibrous encapsulation [1]. As the membranes degraded from day 7 to 14, the reactive capsules thinned, suggesting the membranes’ generally biocompatible nature during degradation.


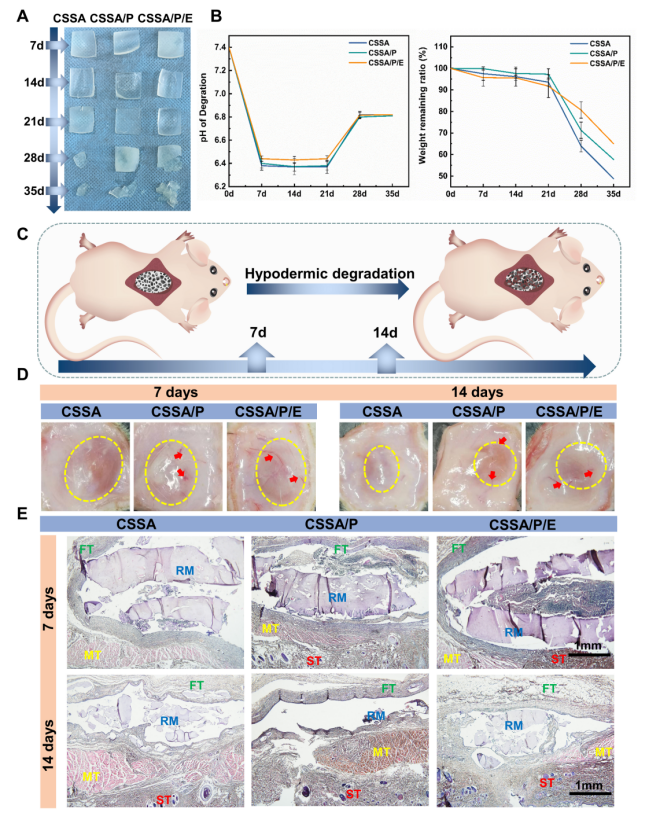


**Figure S5.** Assessment of *in vitro and vivo* degradation of the composite membranes. (A) Morphological change of the composite membranes during degradation; (B) Weight remaining percentage and the pH values during *in vitro* degradation of the composite membranes; (C) Schematic diagram for the *in vivo* degradation test of the composite membranes; (D) Representative gross observation of the subcutaneously implanted composite membranes of 7 and 14 days. The yellow circles indicate areas containing membrane materials, and the red arrows show the blood vessels; (E) The H&E staining after *in vivo* implantation of the composite membranes for 7 and 14 days (bars = 1mm; FT: fibrous tissue; RM: residual membrane; MT: muscle tissue; ST: skin tissue).

**7. *In vivo* biosafety analysis**

To assess the in vivo biological safety of the composite membranes, T-lymphocyte and Pan-macrophage markers were examined. The SABC (Rabbit IgG)-POD Kit was applied for immunostaining for CD3, CD208 and CD86. Positive expressions for CD3, CD206 and CD86 were observed using a light microscope.

**Results analysis**

The initial inflammatory response triggered by material implantation is critical to bone repair. Excessive or prolonged inflammatory responses often result in bone nonunion [7]. Immune cells, which contain macrophages and lymphocytes, are critical to inhibit foreign body reaction and promote bone regeneration [8]. Accordingly, macrophage polarization and lymphocyte expression in subcutaneous implant area were assessed using immunohistochemical staining at 7 and 14 days. Figure S6 shows that the expression of CD86 (M1 marker) on day 7 was significantly higher than that on day 14, indicating a strong early inflammatory response that diminished over time. M2 macrophages, together with T lymphocytes, play important roles in maintaining tissue integrity and immune function. The expressions of CD3 and CD206 increase over time, which could be attributed to the positive correlation between T cell numbers and M2 polarization [9]. Significant CD206 and CD3 expressions triggered by the materials indicate that the composite membrane had immunomodulatory effect and can inhibit the inflammatory response in the initial stage. Totally, the in vivo results have demonstrate the excellent biosafety of the composite membranes.


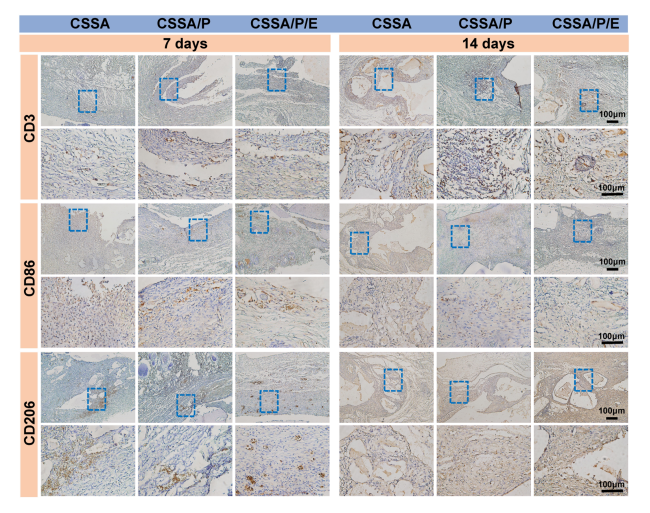


**Figure S6.** Immunohistochemical staining of CD3, CD86, and CD206 at 7 and 14 days of *in vivo* degradation (bars = 100 μm).

**8.Changes in the position of the membrane in the defect area 7 days after implantation**

The rat skull calvarial defect model was used in this experiment. These rats were randomly divided into three groups to implant the composite membranes of CSSA, CSSA/P, or CSSA/P/E, respectively. The position of the material at the defect was observed after 0 and 7 days of implantation, respectively. As shown in Figure S7, the membrane still covered the defect 7 days after implantation.


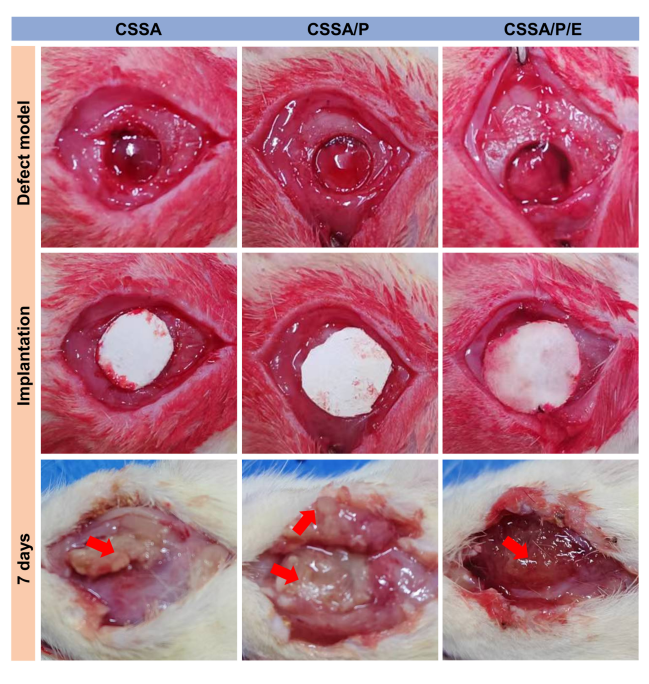


**Figure S7.** The position changes of the material after implantation 0 and 7 days.

**9. *In vivo* cell recruitment early after implantation**

The rats were euthanized with CO_2_ to harvest the skulls with soft tissue 7 days post-surgery for immunohistochemical staining. To evaluate the expression of CD29, CD90 and VEGFR-2, SABC (Rabbit IgG)-POD Kit was applied for immunohistochemical staining. Histological analysis was conducted to observe the distribution of MSCs in the defect under a light microscope.

**Results analysis**

CD90 and CD29 are both important markers for the identification of MSCs, with CD90 linked to cell adhesion, differentiation, cell-cell interaction, and neovascularization formation [10, 11]. The expression of CD29 is positively correlated with the migration of MSCs [12]. On day 7 post-implantation, immunohistochemical staining of CD29 and CD90 was conducted to assess MSCs recruitment to the defect area. Since CD90 itself does not specifically mark endothelial cells or newly formed vessels, it is used in conjunction with VEGFR-2 to accurately assess neovascularization. As illustrated in Figure S8, compared with the control group that was negative for CD90 and CD29, CSSA, CSSA/P and CSSA/P/E exhibited varying degrees of positive staining for CD29 and CD90, demonstrating that they have MSCs recruiting capability. Especially, the rates for the CD29 and CD90 positive staining followed the rank of CSSA/P/E > CSSA/P > CSSA. These results indicated that the membranes’ specific porous structure and the E7 peptide both are instrumental in recruiting MSCs. The high expression of CD90 induced by the membranes may also indicate that recruited MSCs possessed a strong potential for inducing angiogenesis at an early stage, and the high expression of CD29 could display the strong migration capability of these cells. To accurately assess neovascularization, VEGFR-2 was examined. Compared with the control group and CSSA that was negative for VEGFR-2, CSSA/P and CSSA/P/E exhibited varying degrees of positive staining for VEGFR-2, demonstrating that they have neovascularization improving capability.


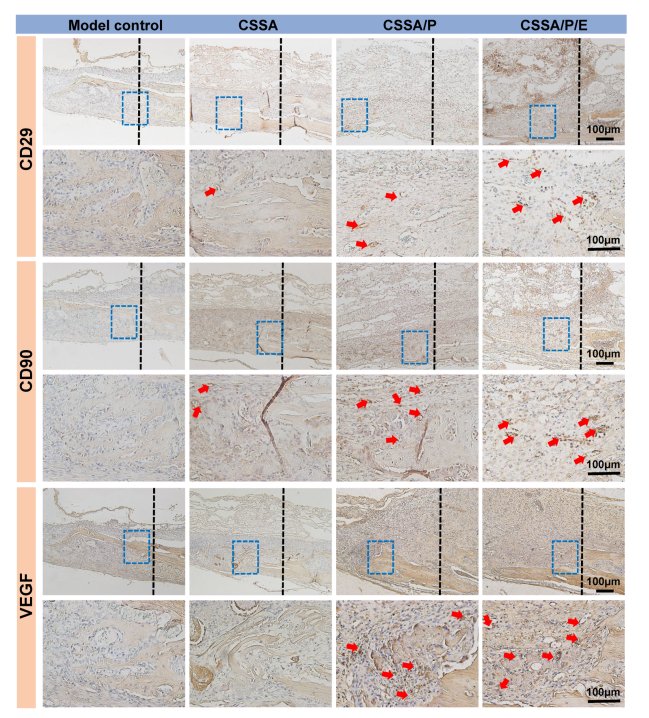


**Figure S8.** Immunohistochemical staining of CD29, CD90 and VEGFR-2 after 7 days implantation of the composite membranes in the rats to show the recruitment of MSCs. (bars = 100 μm; red arrows: MSCs and blood vessel marker; blue dashed boxes: enlarged image).

**10. *In vivo* histologic and blood biochemical analyses of important organs**

Eight weeks post-implantation, SD rats were were euthanized using CO_2_, and major organs including heart, liver, spleen, lung and kidney were harvested. After fixation in 10% formalin for 48 h, the organs were were sectioned, dehydrated, embedded in paraffin, and prepared into 5-μm slices. The paraffin sections were stained with H&E staining and observed under a light microscope.

Four weeks post-implantation, SD rats were anesthetized with an intraperitoneal injection of 0.7 mL 3% pentobarbital solution and secured on the operating table. Blood samples were collected from tail vein and centrifuged at 3000 × g for 10 min to obtain serum after standing for about 1 h. Biochemical analyzer (TBA40FR, Japan) was performed to monitor liver function (e.g., ALT, AST, bilirubin) and kidney function (e.g., creatinine, urea). These procedures were carried out in a dark environment.

**Results analysis**

Since the metabolites produced during the degradation of materials might affect the safety of materials *in vivo*, histological staining of important organs was performed to evaluate the effect of metabolites on the body. Histological examinations of the heart, liver, spleen, lung, and kidney 8 weeks post-implantation showed no obvious morphological or structural changes compared to control group (Figure S9A), confirming the biological safety of the composite membranes. The liver and kidneys, critical for metabolism and detoxification, may be affected by substances released from the interaction of implant materials with surrounding tissues and body fluids. Therefore, assessing liver and kidney function is an essential aspect of evaluating the biocompatibility of implanted materials. Elevated levels of alanine aminotransferase (ALT), aspartate aminotransferase (AST) and total bilirubin (TBIL) in the blood are key indicators of liver dysfunction. Reference ranges for ALT, AST, and TBIL in Sprague-Dawley (SD) rats are reported as 6-114 U/L, 37-205 U/L [13] and 0-5.13 μmol/L [14], respectively. As depicted in Figure S9B, ALT, AST and TBIL levels in all groups fell within acceptable ranges, suggesting no adverse effects on liver function by the composite membrane. Creatinine and blood urea are significant biomarkers for detecting renal dysfunction. The reference ranges for these markers in SD rats are reported as 22.1-73.37 μmol/L for creatinine (Cre-P) [15] and 4.32-8.97 mmol/L for urea [16]. Figure S9C illustrates that the Cre-P and urea values for each group were within normal ranges, indicating no significant impact on renal function by the composite membrane. Overall, these findings suggest that the composite membrane possesses high biocompatibility.





**Figure S9.** (A) H&E staining of major organs in rat 8 weeks after implanting the composite membranes (10x: 250 μm; heart, liver, soleen, lung and kidney); (B) Biochemical analyses of liver and kidney functions (ALT, alanine transaminase; AST, aspartate transaminase; Cre-P, creatinine; UREA, blood urea; TBIL, total bilirubin; n = 3). Data are presented as mean ± SEM. One-way ANOVA was used to calculate statistical signiﬁcance in B-C.

**11. *In vivo* pH environment variation in defect area**

After being sacrificed, the suture line was cut to expose the defect area. The pH of body fluid was measured using universal indicator paper. Simultaneously, the residual implanted materials were retrieved and soaked in PBS solution. After an hour of incubation, a pH meter was used to measure the pH of the PBS solution.

**Results analysis**

As can be seen from Figure S10A, the pH test strips at the three time points were all green, representing a pH range of 7-8. As shown in Figure S10B, the overall pH values remained stable and neutral, which was closely related to the rat body’s intrinsic ability to regulate pH [3].





**Figure S10.** Gross observation (A) and pH value around the defect area 7, 14, and 30 days after implantation.

**References**
